# Winning by Losing: Exploiting Modified Plant Susceptibility Genes to Counteract Necrotrophic Fungal Pathogens

**DOI:** 10.1111/pbi.70331

**Published:** 2025-08-25

**Authors:** Yaohua You, Miguel Ramírez Gaona, Yuling Bai, Jan A. L. van Kan, Anne‐Marie A. Wolters

**Affiliations:** ^1^ Plant Breeding Wageningen University & Research Wageningen Wageningen the Netherlands; ^2^ Institute for Biology 3 RWTH Aachen University Aachen Germany; ^3^ Laboratory of Phytopathology Wageningen University & Research Wageningen the Netherlands

**Keywords:** Arabidopsis, crop plant, host susceptibility, necrotrophic fungus, plant breeding, plant‐microbe interactions, resistance, *S*‐gene

## Abstract

The infection of plants by pathogens is an intricate process in which genes from both the host and pathogen contribute to the infection process. Susceptibility (S) genes have been defined as plant genes that encode functions that are exploited by pathogens to invade and reproduce in host plants. Mutations in *S*‐genes therefore result in reduced susceptibility to the pathogen. The identity and mode of action of *S*‐genes in interactions of plants with biotrophic fungi have been studied since the cloning of the barley *mlo* gene in the late 1990s. The infection strategy of necrotrophic fungi, however, substantially differs from that of biotrophic fungi and therefore is likely to be facilitated by distinct physiological processes in a host plant. There is a rapidly increasing amount of information about *S*‐genes in plants that facilitate the infection by necrotrophic fungi and their mode of action. In this review, we summarise the current knowledge on plant *S‐*genes for susceptibility to necrotrophic fungi and categorise them based on cellular compartments and physiological processes. With recent examples, we subsequently discuss the challenges and opportunities of exploiting impaired plant *S‐*genes for breeding crops with disease resistance.

## 1 Introduction

In contrast to biotrophic pathogens that thrive on living host cells, necrotrophic pathogens kill their host plant cells and then feed on the dead tissues. Instead of indiscriminately killing host cells, they manipulate the plant programmed cell death machinery by first suppressing a cell death response that would result in immunity and subsequently triggering a cell death program that results in host plant susceptibility (Veloso and van Kan 2018). Necrotrophic fungal pathogens cause severe economic losses worldwide and undermine global food security (Fones et al. 2020; Shao et al. 2021). Necrotrophic fungi can be classified based on their host range. For example, fungal pathogens of the class of *Dothideomycetes*, such as *Pyrenophora tritici‐repentis* causing tan spot disease in wheat ( *Triticum aestivum* L.), have a narrow host range (NHR) (Faris and Friesen 2020). In contrast, other necrotrophic fungal pathogens, particularly fungi from the order *Helotiales*, have a broad host range (BHR) (van Kan 2006). For instance, the best‐studied necrotrophic pathogen *Botrytis cinerea* can cause grey mould disease in more than 1500 plant species including both dicots and monocots (Elad et al. 2016). The interaction between *B. cinerea* and its host plants is determined by the balancing of resistance and susceptibility mechanisms in a quantitative manner, resulting in a greyscale of disease outcomes (Veloso and van Kan 2018). Quantitative resistance to BHR necrotrophic fungi is often conferred by multiple quantitative trait loci (QTL) for which the underlying molecular mechanisms are poorly understood (Ding et al. 2021; Finkers, van den Berg, et al. 2007; Finkers, van Heusden, et al. 2007; Finkers et al. 2008; Petrasch et al. 2019, 2022; Rowe and Kliebenstein 2008). This limits the transfer of resistance traits from wild relatives to cultivated plant species. For instance, breeding for resistance against *Alternaria solani* causing early blight disease in tomato ( *Solanum lycopersicum* ) is hampered due to the lack of QTLs providing sufficiently strong resistance (Adhikari et al. 2017; Chaerani and Voorrips 2006).

An alternative approach to breeding for resistance against necrotrophic pathogens starts with the identification of plant susceptibility (*S*) genes that facilitate pathogen infection. The concept of *S‐*gene utilisation in breeding for resistance to (hemi)biotrophic pathogens has been reviewed by several authors (van Schie and Takken 2014; Garcia‐Ruiz et al. 2021; Koseoglou et al. 2022; van Damme et al. 2023), a comprehensive overview of *S‐*genes for necrotrophic fungal pathogens is thus far lacking. A rapidly increasing number of *S*‐genes for such pathogens has been reported in plants, notably in Arabidopsis (Table S1), and they provide promising targets for breeding because their disruption can lead to reduced plant susceptibility. In this review, we focus on insights into *S*‐gene‐mediated susceptibility and the perspectives of utilising impaired *S‐*genes in breeding crops with resistance to necrotrophic pathogens.

## 2 *S*‐Genes: A Single Term Comprising Diverse Functions

Unlike the majority of plant resistance (*R*) genes that encode cell surface or intracellular receptors (Kourelis and van der Hoorn 2018), *S*‐genes mediate diverse functions and participate in the plant‐microbe interactions in distinct manners. Based on the susceptibility mechanisms of more than 100 cloned *S*‐genes whose functional disruption leads to reduced susceptibility to necrotrophic fungal pathogens, we distinguish *S*‐genes into six categories that are associated with different fungal infection stages and/or distinct cellular processes in the plant. These six categories are discussed in the next sections. The genes which have been reported to act as *S*‐genes for necrotrophic fungi in Arabidopsis are provided in Table S1, while *S‐*genes reported for crop plants are listed in Table 1, sorted by the functional category to which we assigned them.

**TABLE 1:** Information of *S*‐genes mediating susceptibility to necrotrophic fungal pathogens identified in crop plants.

Protein/molecule	Gene	Host	Pathogen(s)	Function	References
*Plant cell wall remodelling*					
Ferulate‐5‐hydroxylase	*F5H*	Rapeseed	*Sclerotinia sclerotiorum*	Converts guaiacyl monolignol to syringyl monolignol	Cao, Yan, et al. (2022)
Pectate lyase	*PL*	Tomato	*B. cinerea*	PCWDE, cleaves α‐1,4 glycosidic bonds of pectate by ß‐elimination	Silva et al. (2021); Yang et al. (2017)
—	*TBL*	Rose	*B. cinerea*	Likely mediates PCW acetylation	Tian et al. (2021)
Polygalacturonase	*PG*	Tomato	*B. cinerea*	PCWDE, hydrolyzes α‐1,4 glycosidic bonds of pectin	Cantu et al. (2008)
Expansin	*Exp1*	Tomato	*B. cinerea*	PCW‐loosening agent	Cantu et al. (2008)
Polygalacturonase	*PG1*	Strawberry	*B. cinerea*	PCWDE, hydrolyzes α‐1,4 glycosidic bonds of pectin	López‐Casado et al. (2023)
Endo‐b‐1,4‐glucanase	*Cel1*, *Cel2*	Tomato	*B. cinerea*	PCWDE, exact functions await elucidation	Flors et al. (2007)
Glucosyltransferase	*UGT88F1*	Apple	*Valsa mali*	Catalyses phloridzin biosynthesis	Zhou et al. (2019)
*Plant cell death regulation*					
Histidine rich Ca^2+^ binding protein	*HRC*	Potato	*Alternaria solani*	Participates in induction of apoptotic‐like PCD in plant	Kushalappa et al. (2022)
U‐box E3 ubiquitin ligase	*PUB17*	Tomato	*Botrytis cinerea, A. solani*	Participates in plant PCD in response to biotroph	Ramírez Gaona et al. (2023); Yang et al. (2006)
α/β hydrolase‐fold family protein	*NYC3*	Rice, maize	*Rhizoctonia solani*	Mediates chlorophyll degradation, participates in PCD	Cao, Zhang, et al. (2022)
Metacaspase	*MC8*	Tomato	*A. solani*	Contributes to ROS accumulation and promotes PCD in plant	Basak et al. (2024)
Cyclic nucleotide‐gated ion channel	*DND1*	Tomato	*B. cinerea*	Mediates Ca^2+^ influx into cytosol and participates in induction of PCD in plant	Sun et al. (2017)
Hypersensitive‐induced reaction protein	*HIR2*	*Nicotiana benthamiana*	*S. sclerotiorum*	Interacts with fungal xylanase and promotes its plant PCD inducing activity	Wang et al. (2024)
—	*SGT1*	*N. benthamiana*	*B. cinerea*	Participates in plant PCD in response to biotrophs	El Oirdi and Bouarab (2007)
Lipase	*EDS1*	*N. benthamiana*	*B. cinerea*	Participates in plant PCD in response to biotrophs	El Oirdi and Bouarab (2007)
NLR protein	*Pc*	Sorghum	*Periconia circinata*	Required for host sensitivity to PC‐toxin	Nagy and Bennetzen (2008); Nagy et al. (2007)
NLR protein	*Tsn1*	Common wheat	*Parastagonospora nodorum, Pyrenophora tritici‐repentis, Bipolaris sorokiniana*	Required for host sensitivity to ToxA	Faris et al. (2010)
Wall‐associated kinase	*Snn1*	Common wheat	*P. nodorum*	Required for host sensitivity to SnTox1	Shi et al. (2016)
—	*T‐urf13*	Maize	*Cochliobolus victoriae*	Required for host sensitivity to T‐toxin	Rhoads et al. (1995)
Serine/threonine protein kinase	*Snn3‐D1*	Common wheat	*P. nodorum*	Required for host sensitivity to SnTox3	Zhang, Running, et al. (2021)
*Fungal nutrient availability*					
Sugar transporter	*SWEET15*	Soybean	*S. sclerotiorum*	Exact functions await elucidation	Xiao et al. (2023)
Sugar transporter	*SWEET2a*, *SWEET3a*	Rice	*R. solani*	Hexose transporter, induced by fungal AOS2 protein to excrete sugars from the cytosol to the apoplast	Yang et al. (2023)
Sugar transporter	*SWEET11*	Rice	*R. solani*	Hexose transporter, excrete sugars from the cytosol to the apoplast	Gao et al. (2018)
*Crosstalk of phytohormone‐mediated signalling pathways and suppression of basal immunity*					
—	*NPR1*	Tomato	*B. cinerea*	Positively regulates SA signalling, negatively regulates JA‐mediated defences	El Oirdi et al. (2011); Ndamukong et al. (2007)
ABA‐aldehyde oxidase	*AAO*	Tomato	*B. cinerea*	Involved in ABA synthesis, converts ABA‐aldehyde to ABA	Audenaert et al. (2002)
TGA TF	*TGA1.a*	Tomato	*B. cinerea*	Negatively regulates the expression of JA‐mediated defence genes *PI I* and *PI II*	Ekengren et al. (2003); Rahman et al. (2012)
Phosphatidylinositol‐phospholipase	*PLC2*	Tomato	*B. cinerea*	Repressor of JA‐mediated defences	Gonorazky et al. (2016); Perk et al. (2023)
B‐box TF	*BBX20*	Tomato	*B. cinerea*	Interacts with MED25 and prevents the activation of JA‐mediated defence genes	Luo et al. (2023)
Receptor‐like cytoplasmic kinase	*Fir1*	Tomato	*B. cinerea*	Interacts with flagellin receptors Fls2 and Fls3, negatively regulates JA‐mediated defences	Sobol et al. (2023)
12‐oxo‐phytodienoic acid reductase	*OPR2*	Maize	*C. heterostrophus*	Negatively regulate JA biosynthesis	Huang et al. (2023)
Cytochrome P450 oxidase	*CYP94C1*	Tomato	*B. cinerea*	Converts JA‐Ile to its inactive form 12‐COOH‐JA‐Ile, terminates JA signalling	Yang, Deng, et al. (2024)
WRKY TF	*WRKY70*	Rapeseed	*S. sclerotiorum*	Represses the expression of SARD1 to supress SA‐mediated defences	Sun et al. (2018); Zhou et al. (2018)
WRKY TF	*WRKY29*, *WRKY64*	Strawberry	*B. cinerea*	Regulates ABA and JA signalling	Lee, Han, and Lee (2023)
WRKY TF	*WRKY3*	Tomato	*B. cinerea*	Regulates SA signalling, binds to the promoter of *TPK1b* and negatively regulates its transcription	Luo et al. (2024)
RLK	*RLK902*	Rapeseed	*S. sclerotiorum, B. cinerea*	Likely negatively regulates JA‐mediated defences	Zhao et al. (2024)
TF	*EIL3*	Rice	*R. solani*	Negatively regulates SA‐ and JA‐ mediated defences	Zhu et al. (2024)
WRKY TF	*WRKY1*	Chinese cabbage	*B. cinerea*	Inhibits JA biosynthesis	Yuan et al. (2024)
Cytokinin dehydrogenase	*CKX3*	Rose	*B. cinerea*	Catalyses the degradation of cytokinin	Liu et al. (2023)
AP2 TF	*ABI4*	Rose	*B. cinerea*	Positively regulates ABA signalling	Liu et al. (2023)
TF	ARF8	Tomato	*B. cinerea, A. alternata*	Regulates auxin signalling	Marash et al. (2024)
F‐box protein	*FBL41*	Maize	*R. solani*	Mediates the degradation of ABA synthase	Yang, Liang, et al. (2024)
RLK	*ChSK1*	Maize	*C. heterostrophus*	Modulates ROS production and affects plant defence response	Chen et al. (2023)
C2 domain Ca^2+^ sensor	*ROD1*	Rice	*R. solani*	Promotes ROS scavenging by stimulating catalase activity	Gao et al. (2021)
*Epigenetic regulation and post‐transcriptional silencing*					
Microrchidia protein	MORC1, MORC6a	Barley	*Bipolaris sorokiniana, Fusarium graminearum*	Negatively regulates plant immunity, interacts with components of the RdDM pathway.	Galli, Hochstein, et al. (2022); Galli, Martiny, et al. (2022)
Histone H3 lysine methyltransferase	SDG33, SDG34	Tomato	*B. cinerea*	Mediate methylation of H3K4 and H3K36.	Bvindi et al. (2022)
Argonaute protein	AGO18b	Maize	*C. heterostrophus*	Component of RNA‐induced silencing complex	Dai et al. (2023)

Abbreviations: ER, endoplasmic reticulum; HR, hypersensitive response; NLR, nucleotide‐binding domain, leucine‐rich repeat containing protein; PCD, programmed cell death; PCW, plant cell wall; PCWDE, plant cell wall degrading enzyme; PM, plasma membrane; RdDM, RNA‐directed DNA methylation; RLK, receptor‐like kinase; ROS, reactive oxygen species; TF, transcription factor.

## 3 *S*‐Genes Involved in Cuticle Formation: Broken Isn't Always Bad

**FIGURE 1:**
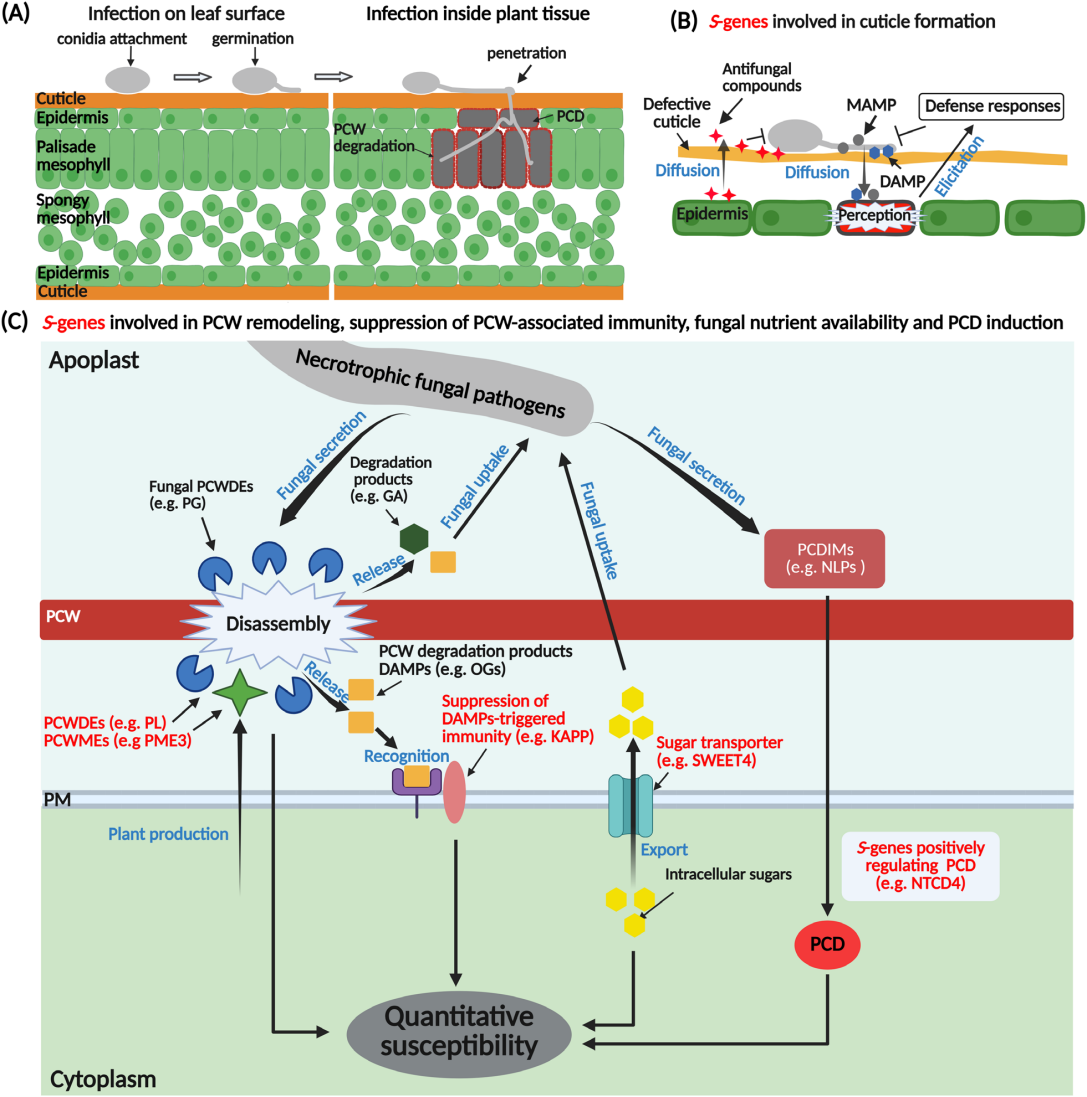
(A) Schematic illustration of the infection process of necrotrophic fungal pathogens starting from the attachment of a spore on the plant leaf surface. (B) *S*‐genes involved in cuticle formation. (C) *S*‐genes involved in PCW remodelling, suppression of PCW‐associated immunity, fungal nutrient availability and PCD induction. Proteins encoded by *S*‐genes are marked in red. T‐shaped lines indicate inhibition. Detailed information on these types of *S*‐genes is provided in Table 1 and Table S1. DAMP, damage‐associated molecular pattern; GA, galacturonic acid; KAPP, kinase‐associated protein phosphatase; MAMP, microbe‐associated molecular pattern; NLP, necrosis‐ and ethylene‐inducing‐like protein; NTCD4, NLP‐triggered cell death on chromosome 4; OGs, oligogalacturonides; PCD, plant cell death; PCDIM, plant cell death inducing molecule; PCW, plant cell wall; PCWDE, plant cell wall‐degrading enzymes; PCWME, plant cell wall‐modifying enzyme; PG, polygalacturonase; PL, pectate lyase; PM, plant membrane; PME3, pectin methylesterase 3; SWEET4, sugars will eventually be exported transporter 4. Created in https://BioRender.com. Bai (2025) https://BioRender.com/k3laoon.

Infection of necrotrophic fungal pathogens on aerial parts of plants such as leaves, stems and flowers starts from the attachment of spores to the plant surface followed by spore germination, elongation of germ tubes and growth of mycelium (Bi et al. 2023) (Figure 1A). The outermost cell layer of plant tissues (epidermis) is covered with a cuticle, a polymeric matrix composed of cutin (polyhydroxylated fatty acids cross‐linked by ester bonds) and waxes (Domínguez et al. 2011; Heredia 2003). The aliphatic and hydrophobic cuticular layer serves as a barrier between plant and ambient environment and it plays an important role in (a)biotic stress tolerance as well as plant development (Domínguez et al. 2017; Serrano et al. 2014). Necrotrophic fungal pathogens often form specialised structures known as appressoria (unicellular) or infection cushions (multicellular), which secrete hydrolytic enzymes such as cutinases and lipases to degrade and penetrate the plant cuticle (Arya and Cohen 2022; Choquer et al. 2021).

Mutation of genes required for the synthesis of cutin and wax, such as Long‐chain acyl‐CoA synthetase *LACS2* (Tang et al. 2007), ABC transporter *ABCG32* (Bessire et al. 2011) or transcription factor *MYB96* (Benikhlef et al. 2013) leads to loss of cuticle integrity and increased permeability (Figure 1B). Interestingly, such cuticular defects conferred strong resistance to necrotrophic fungal pathogens including *B. cinerea* and *Sclerotinia sclerotiorum*, manifested as inhibition of spore germination and hyphal elongation on the leaf surface (Chassot et al. 2007; Tang et al. 2007; Uppalapati et al. 2012). Strong antifungal activity was detected in the leaf surface diffusate of these Arabidopsis mutants, potentially due to increased cuticle permeability that allows the diffusion of uncharacterized fungitoxic compounds (Bessire et al. 2007; Serrano et al. 2014; Ziv et al. 2018). Increased cuticle permeability may also facilitate the diffusion into the host tissue of fungal molecules known as pathogen‐associated molecular patterns (PAMPs) and damage‐associated molecular patterns (DAMPs) derived from cuticle degradation by the fungus. Both PAMPs and DAMPs can be perceived by host plant cells and induce defence responses (Aragón et al. 2017; Serrano et al. 2014; Ziv et al. 2018). In addition, the constitutive production of reactive oxygen species (ROS) was detected in Arabidopsis cuticle mutants *bodyguard* (*bdg*) and *lacs2* before inoculation (L'Haridon et al. 2011). The constitutive activation of plant defences in cuticle mutants might be attributed to the excessive accumulation of biosynthetic intermediates of cuticle constituents, such as cutin monomers, which can trigger plant defence responses (Serrano et al. 2014). Moreover, resistance to necrotrophic fungal pathogens caused by cuticular defects was also found to be mediated by the phyllosphere microbiome, as reported in Arabidopsis *bdg* mutants whose leaf washes had distinct bacterial communities as compared to wild‐type (WT) plants (Ritpitakphong et al. 2016). Application of phyllosphere microbes extracted from *bdg* mutants to the WT plants increased resistance to *B. cinerea* , whereas the *bdg* mutant grown under sterile conditions became equally susceptible to *B. cinerea* as WT plants (Ritpitakphong et al. 2016).

Overall, the studies on the role of cuticle defects in reducing susceptibility to necrotrophs are limited to Arabidopsis (Table S1). Studies in tomato revealed that the role of the cuticle in conferring susceptibility to necrotrophs might be tissue‐specific. Reduced susceptibility to *B. cinerea* associated with rapid activation of defence responses was observed in the leaves of tomato cuticle mutant *sitiens* (Asselbergh et al. 2007; Curvers et al. 2010). However, the fruit of tomato cuticle mutants *cd1*, *cd2* and *cd3* displayed increased susceptibility to *B. cinerea* (Isaacson et al. 2009).

## 4 *S*‐Genes Related to Plant Cell Wall Remodelling: Unbreakable Is the Key

The plant cell wall (PCW) is a complex and dynamic matrix and constitutes an important physical barrier against the invasion of (necrotrophic) pathogens (Molina et al. 2021; Wan et al. 2021). Necrotrophic fungi secrete PCW‐degrading enzymes such as polygalacturonases as important virulence factors to decompose PCW and provide nutrients for fungal biomass accumulation (Figure 1C) (Bi et al. 2023; Liao et al. 2022). Moreover, the action of plant‐derived PCW hydrolases and modifying proteins can confer susceptibility to necrotrophic fungi (Bandara et al. 2018; Bellincampi et al. 2014; Kubicek et al. 2014). For instance, *B. cinerea* can facilitate PCW disassembly through the acceleration of tomato fruit ripening, including the induction of tomato pectate lyase (*PL*) gene expression (Silva et al. 2021). Notably, disruption of *PL* in tomato delayed fruit softening and reduced susceptibility to *B. cinerea* (Silva et al. 2021; Yang et al. 2017). In Arabidopsis, induction of pectin methylesterase 3 (*PME3*) mediating PCW modification during necrotrophic infection contributes to host susceptibility (Raiola et al. 2011). Plants can monitor their cell wall integrity through the sensing of PCW degradation products serving as DAMPs. The best‐studied DAMPs are the oligogalacturonides (OGs) released by hydrolysis of homogalacturonan (Ishida and Noutoshi 2022; Pontiggia et al. 2020). OGs can be perceived by plant pattern‐recognition receptors (PPRs) which subsequently trigger defence responses (De Lorenzo and Cervone 2022). Remarkably, plants can also quench DAMP‐triggered plant immunity (Huot et al. 2014). In Arabidopsis, a glycine‐rich protein (GRP‐3) and a kinase‐associated protein phosphatase (KAPP) on the plasma membrane were found to suppress the OG‐triggered defence responses and thereby increased plant susceptibility to *B. cinerea* (Gramegna et al. 2016).

Several cellulose synthesis (*CESA*) genes are involved in secondary plant cell wall biosynthesis. Disruption of the *CesA4* gene, or its reduced expression caused by mutation of transcription factor *MYB46*, led to increased ABA levels as well as constitutive expression of ABA‐responsive antimicrobial peptides (Hernández‐Blanco et al. 2007; Ramírez et al. 2011). This resulted in increased resistance to *B. cinerea* and *Plectosphaerella cucumerina*.

## 5 *S*‐Genes Regulating Plant Cell Death: The Suicide Squad

The plant cell death (PCD) induction by necrotrophic pathogens often displays hallmarks of programmed cell death resembling apoptosis (Figure 1A) (Kim et al. 2008; Van Baarlen et al. 2004). NHR necrotrophic fungal pathogens produce host‐selective toxins (HSTs) which often exert toxicity on a limited number of plant species (Kanyuka et al. 2022; Laluk and Mengiste 2010; Stergiopoulos et al. 2013; Wolpert et al. 2002). So far, several plant genes have been identified that encode cell surface or intracellular receptors which directly or indirectly interact with HSTs and thereby confer susceptibility to NHR necrotrophic fungi. Remarkably, the majority of these *S*‐genes display sequence similarity to gene families conferring resistance to biotrophic pathogens (Faris and Friesen 2020; Liao et al. 2022). For instance, *P. tritici‐repentis* secretes a 13‐kDa ToxA protein that mediates disease in wheat (Ciuffetti et al. 2010). Sensitivity to ToxA and susceptibility to *P. tritici‐repentis* is governed by the wheat *Tsn1* gene which encodes a protein containing serine/threonine protein kinase (S/TPK), nucleotide binding site (NBS) and leucine‐rich repeat (LRR) domains (Faris et al. 2010).

In contrast, BHR necrotrophic fungal pathogens such as *B. cinerea* produce an array of molecules including proteins, secondary metabolites and ROS that can induce PCD in multiple plant species (Bi et al. 2023; Leisen et al. 2022; Liao et al. 2022; Newman et al. 2023). Arabidopsis *DND1* (defence, no death1), encoding a cyclic nucleotide‐gated ion channel (CNGC) protein, was the first *S*‐gene that was reported to confer susceptibility to BHR necrotrophic fungal pathogens through regulating PCD (Clough et al. 2000; Govrin and Levine 2000). Later on, a number of genes positively regulating PCD through diverse mechanisms have been demonstrated to function as *S*‐genes to BHR necrotrophic pathogens (Figure 1C, Table 1). A resistance‐like gene, *LAZ5* (*Lazarus5*) in Arabidopsis encodes an NBS‐LRR protein that positively regulates PCD and thereby facilitates disease progression of *S. sclerotiorum* (Barbacci et al. 2020). Another LRR protein, NTCD4 (NLP‐Triggered Cell Death on chromosome 4), promoted the cytolytic activity of *B. cinerea* NLPs (necrosis‐ and ethylene‐inducing‐like proteins) by assisting their oligomerization in the apoplast (J.B. Chen et al. 2021). Our recent study identified a nuclear U‐box E3 ubiquitin ligase PUB17 in tomato, known to be required for a hypersensitive response (HR) induced by the biotrophic pathogen *Cladosporium fulvum*, that serves as an *S*‐gene to *B. cinerea* and *A. solani* (Ramírez Gaona et al. 2023; Yang et al. 2006).

Interestingly, the LRR receptor‐like kinase (RLK) BAK1 was recently reported to repress autophagy by phosphorylation of autophagy‐related protein 18a (ATG18a) and promote susceptibility to *B. cinerea* (Zhang, Shao, et al. 2021). This observation corroborates the model proposed by Veloso and van Kan (2018) that BHR necrotrophic pathogens suppress autophagy in an early stage of infection to avoid plant resistance and, later on, induce apoptosis to cause expanding necrotic lesions.

## 6 *S*‐Genes Facilitating Fungal Nutrient Availability: Stop Feeding the Enemies

SWEET (Sugars Will Eventually be Exported Transporters) proteins in the plasma membrane mediate the cellular export of sugars and thereby facilitate fungal nutrient acquisition and promote host susceptibility (Figure 1C) (Chen et al. 2010, 2024; Gupta 2020; Liu, Song, et al. 2022). It has been reported that necrotrophic fungal pathogens take advantage of plant sugar transport via induction of *SWEET* genes to facilitate infection; whereas host susceptibility was reduced when *SWEET* genes were disrupted (Liu, Song, et al. 2022). In Arabidopsis, transcripts of several *SWEET* genes were induced by *B. cinerea* and the mutation of *SWEET4* resulted in increased resistance to *B. cinerea* (Chen et al. 2010; Chong et al. 2014). Moreover, a recent study also showed that loss of function of *SWEET15a* in soybean ( *Glycine max* ) led to reduced susceptibility to *S. sclerotiorum* (Xiao et al. 2023).

## 7 *S*‐Genes Regulating Hormone Signalling and Crosstalk: Choosing the Right Armour

Phytohormones are signalling molecules controlling many aspects of a plant life cycle (Berry and Argueso 2022; Kim et al. 2022; Verma et al. 2016). Different phytohormone‐mediated signalling pathways can work antagonistically and cooperatively in a regulatory network referred to as hormone crosstalk, which not only modulates plant immunity but also regulates the growth–defence trade‐off (He et al. 2022; Huot et al. 2014; Shigenaga et al. 2017).

Studies on plant vegetative tissues revealed that jasmonic acid (JA) and ethylene (ET) signalling pathways synergistically activate plant defence against necrotrophic pathogens, while salicylic acid (SA) signalling is mainly involved in resistance to biotrophic pathogens (Glazebrook 2005; Kim et al. 2022). Although genes involved in hormonal pathways are usually considered to play a role in resistance, they may also be hijacked by necrotrophic pathogens in order to cause cell death. In that respect, they may also be considered as *S*‐genes for this group of pathogens.

Negative regulators of JA‐ and ET‐mediated defence were found to promote necrotrophic fungal invasion (Table S1). For example, the SA signalling component Nonexpressor of Pathogenesis‐Related genes1 (NPR1) negatively regulates JA‐mediated defence (Ndamukong et al. 2007; Spoel et al. 2003). Silencing of *NPR1* in tomato partially relieved the suppression of JA‐defence and reduced susceptibility to *B. cinerea* (El Oirdi et al. 2011). These negative regulators also involved a number of transcription factors (TFs) (Table 1 and Table S1). For instance, TFs from the Apetala2/Ethylene Response Factor (AP2/ERF) family including ERF9 and ERF014 were shown to promote susceptibility of Arabidopsis to *B. cinerea* and *A. brassicicola* via attenuating JA‐mediated defence (Maruyama et al. 2013; Zhang et al. 2016). In addition, Arabidopsis WRKY57, a WRKY family TF member, was shown to directly activate genes encoding JASMONATE ZIM‐DOMAIN (JAZ) proteins, which act as repressors of JA signalling, and the loss of function of *WRKY57* enhanced plant resistance to *B. cinerea* (Jiang and Yu 2016). Moreover, susceptibility mechanisms also involve post‐transcriptional regulation. WRKY33 is a key regulator of phytohormone signalling and contributes to plant resistance against necrotrophic fungal pathogens (Xie et al. 2020; Zheng et al. 2006). Notably, a recent study reported that WRKY33‐mediated defence required post‐transcriptional modification by small ubiquitin‐like modifier (SUMO) proteins, whereas deSUMOylation of WRKY33 mediated by SUMO proteases SPF1 and SPF2 resulted in lower phosphorylation status and compromised its transcriptional activity (Verma et al. 2021). Interestingly, this post‐transcriptional modification facilitated necrotrophic infection, as the Arabidopsis *spf1 spf2* double knock‐out (KO) mutants exhibited reduced susceptibility to *B. cinerea* (Verma et al. 2021).

Abscisic acid (ABA) signalling regulates plant cuticle formation and can antagonise JA/ET/SA‐mediated defence; therefore, it plays an important role in plant‐microbe interactions (Cui et al. 2016; Martin et al. 2017). Disruption of ABA biosynthetic genes or signalling components was found to increase resistance to necrotrophic pathogens including *B. cinerea* and *Plectosphaerella cucumerina* in tomato (Audenaert et al. 2002) and Arabidopsis (García‐Andrade et al. 2020; Liu et al. 2015; Sánchez‐Vallet et al. 2012). However, it was also suggested that ABA contributes to resistance against *S. sclerotiorum* and *A*. *brassicicola* (Adie et al. 2007; Guimarães and Stotz 2004; Guo and Stotz 2010).

Gibberellic acid (GA) signalling appears to promote necrotrophic pathogen invasion through negative modulation of JA‐signalling (Shigenaga et al. 2017). This occurs by GA‐mediated degradation of DELLA proteins which orchestrate hormone crosstalk and positively regulate JA‐mediated defence (Hou et al. 2010; Navarro et al. 2008). Corroborating this notion, Arabidopsis KO mutants of *SLY1‐10* (*SLEEPY1*) encoding an F‐box protein positively regulating GA signalling displayed reduced susceptibility to *A. brassicicola* (Navarro et al. 2008).

Studies using Arabidopsis mutants indicated that the auxin signalling pathway plays a positive role in resistance against necrotrophic pathogens (Kazan and Manners 2009; Llorente et al. 2008; Qi et al. 2012). The Arabidopsis *Gretchen Hagen 3* (*GH3*) gene mediating conjugation of indole‐3‐acetic acid (IAA) to aspartic acid (Asp) was strongly upregulated by necrotrophic infection and facilitated infection of *B. cinerea* (González‐Lamothe et al. 2012). Interestingly, the susceptibility‐promoting effect of IAA‐Asp as a biologically inactive derivative seemed to be associated with the induction of fungal virulence genes rather than influencing phytohormone signalling (González‐Lamothe et al. 2012).

It is worth mentioning that the roles of phytohormones in interactions between plants and necrotrophs are tissue‐ and developmental stage‐specific. Most of the studies discussed above are limited to vegetative tissues. However, the production of ripening‐promoting hormone ET in tomato fruit was rapidly induced by *B. cinerea* infection and led to increased susceptibility (Silva et al. 2023).

## 8 *S*‐Genes Involved in Epigenetic Regulation: New Players in the Game

Epigenetic regulation is emerging as an important player in plant‐microbe interactions (Ashapkin et al. 2020; Liao et al. 2022; Parker et al. 2022; Xie and Duan 2023). Recent studies revealed the role of plant epigenetic regulation in promoting susceptibility to necrotrophic pathogens (Table 1, and Table S1). Members of the microrchidia family proteins (MORC1 and MORC6a) mediating chromatin compaction were shown to repress the induction of pathogenesis‐related (*PR*) genes in barley ( *Hordeum vulgare* ) and conferred susceptibility to *Bipolaris sorokiniana* causing *Bipolaris* spot blotch (Galli, Hochstein, et al. 2022; Galli, Martiny, et al. 2022). In addition, *B. cinerea* can induce tomato SET domain protein genes *SDG33* and *SDG34*, encoding histone lysine methyltransferases to manipulate global histone methylation in the plant (Bvindi et al. 2022). The tomato double KO mutants of *SDG33* and *SDG34* were less susceptible to *B. cinerea* . However, the susceptibility mechanisms influenced by these epigenetic modifications await elucidation (Bvindi et al. 2022).

MicroRNAs (miRNAs) are short, non‐coding RNAs that play an important role in post‐transcriptional gene silencing. When miRNAs are loaded into ARGONAUTE1 (AGO1) the resulting miRNA‐induced silencing complex (miRISC) degrades messenger RNA (mRNA) of target genes with complementary sequences. Several miRNAs in Arabidopsis were shown to negatively regulate defence against necrotrophic fungi. For instance, enhanced JA and ET‐mediated defence concurrently with reduced susceptibility to *B. cinerea* and *P. cucumerina* was observed in transgenic Arabidopsis with reduced miR396 activity (Soto‐Suárez et al. 2017). These transgenic plants showed reduced expression of *S*‐gene *Enhanced Disease Susceptibility 1* (*EDS1*) (Soto‐Suárez et al. 2017). Similarly, Arabidopsis miR773 was identified as a negative regulator of callose deposition and induction of both JA‐ and SA‐mediated defence by downregulating its target gene *Methyltransferase 2* (*MET2*) (Li et al. 2010; Salvador‐Guirao et al. 2018). Interference with miR773 or overexpression of *MET2* increased resistance to *P. cucumerina* in Arabidopsis (Salvador‐Guirao et al. 2018). It is noteworthy that fungal small interfering RNAs (siRNAs) can enter host plant cells and be loaded into AGO1 to silence plant immunity genes, and reduced susceptibility to *B. cinerea* was observed in Arabidopsis *ago1* mutants (Weiberg et al. 2013).

## 9 From Fungal Foes to Friendly Fields: Current Status on Resistance Breeding With *S*‐Genes

Silencing or mutation of *S*‐genes in crop plants has been applied to obtain resistance against various pathogens including viruses (e.g., *elF4E* in pepper), pathogenic bacteria (*SWEET* genes in rice), biotrophic fungi (e.g., *MLO* in many plant species) and hemibiotrophic fungi (e.g., *DMR6* in tomato) (Fidan et al. 2023; Jørgensen 1992; Moury et al. 2020; Oliva et al. 2019; Schmitt‐Keichinger 2019; Thomazella et al. 2021). As for combating necrotrophic fungi, wheat cultivars have been produced that carry the impaired *S*‐gene *Tsn1* (See et al. 2024) to control diseases caused by NHR necrotrophic fungi such as *Pyrenophora tritici‐repentis* and *Parastagonospora nodorum* (Oliver et al. 2014; See et al. 2024; Vleeshouwers and Oliver 2014). With the identification of the tomato *S*‐gene *PUB17* for BHR necrotrophic fungi, it is expected that tomato cultivars with resistance to *B. cinerea* and *A. solani* will be released (Ramírez Gaona et al. 2023).

## 10 Gene Treasure Hunt: Searching for *S*‐Genes in Crops

In the model plant Arabidopsis, a large number of *S*‐genes has been identified and characterised (Table S1). To identify and verify their putative orthologs in crop species, reverse genetics can be applied. This involves allele mining of the candidate genes in natural germplasms and mutagenized populations (Tripathi et al. 2023). Accumulating evidence indicates that *S*‐genes are conserved for their *S*‐gene function across plant species. For instance, *DND1* genes in Arabidopsis, potato and tomato were shown to promote susceptibility to *B. cinerea* (Govrin and Levine 2000; Sun et al. 2017). However, exceptions are also known, demonstrating that validation of the function of *S‐*gene homologues in crops is essential. For example, MYC2 acts as a negative regulator of JA‐mediated defence in Arabidopsis and promotes susceptibility *to B. cinerea* (Lorenzo et al. 2004; Nickstadt et al. 2004), but tomato MYC2 was found to positively regulate JA‐mediated defence, and mutation of MYC2 in tomato compromised resistance against *B. cinerea* (Du et al. 2017).

Additionally, in some crop species, a forward genetic approach has been used to identify mutant alleles of *S*‐genes by screening wild germplasm and mutagenized populations for resistant plants (Piechota et al. 2020; Ramírez Gaona et al. 2023). This resulted in the identification of an increasing number of *S*‐genes conferring susceptibility to necrotrophic fungi in crop species (Table 1). With advanced tools for digital phenotyping and large‐scale genotyping, more *S*‐genes may be discovered by genetic mapping approaches. Examples are the *Amino Acid Permease* (*AAP*) genes in tomato and cucumber (Berg et al. 2021). Recently, proximity labelling has been exploited as a tool to identify pathogen effectors targets, which may be encoded by potential *S*‐gene candidates (Shi et al. 2022).

## 11 Side Effects of Editing *S*‐Genes: Harnessing a Double‐Edged Sword

Pleiotropic side effects caused by disruption of *S*‐genes pose a long‐standing concern due to the diverse functions of *S*‐genes that regulate different aspects of plant growth, development and stress responses (van Schie and Takken 2014). For instance, reduced susceptibility to necrotrophs by mutations in *S*‐genes involved in cuticle formation resulted in enhanced cuticle permeability and conferred increased sensitivity to abiotic stresses (drought and hypoxia), dwarfism and abnormal organ formation (Ingram and Nawrath 2017; Lee and Suh 2015; Liu, Wang, et al. 2022). In addition, PCD processes that facilitate necrotrophic infection (Veloso and van Kan 2018) may play a fundamental role in regulating plant development and resistance against biotrophic and hemibiotrophic pathogens (Balint‐Kurti 2019; Pitsili et al. 2020). For example, silencing the SA glucosyltransferase gene *SGT1*, which is required for PCD, improved resistance to *B. cinerea* but reduced resistance to biotrophic pathogens (Azevedo et al. 2002; El Oirdi and Bouarab 2007). Additionally, impairment of S‐genes that recognise PCD‐inducing molecules from necrotrophs could weaken innate immunity, as demonstrated by the silencing of *RE02* in *Nicotiana benthamiana*, which increased susceptibility to *S. sclerotiorum* (Nie et al. 2021). Also, altering *S*‐genes involved in phytohormone‐mediated signalling pathways, particularly those related to abscisic acid (ABA) and gibberellin (GA), can negatively impact plant growth and tolerance to abiotic stress (Berry and Argueso 2022). Similarly, attempts to reduce host susceptibility through interference with epigenetic regulation have shown that this approach can result in adverse effects, as observed in barley *morc1* and *morc6a* mutants, which exhibited increased resistance to necrotrophic fungi at the expense of plant growth (Galli, Hochstein, et al. 2022; Galli, Martiny, et al. 2022).

Fortunately, an increasing number of studies of plant *S*‐genes has revealed that reduced susceptibility through editing *S*‐genes is not necessarily linked to reduced plant fitness. For instance, reduced susceptibility to necrotrophic fungal pathogens via KO of *RLK902* encoding a receptor‐like kinase in rapeseed is not accompanied by growth penalties (Zhao et al. 2024). Mutants of rice *S*‐genes including *SWEET11* and *NYC3* were less susceptible to *R. solani* and did not display yield losses (Cao, Zhang, et al. 2022; Gao et al. 2018). Notably, targeting *S*‐genes associated with PCW modification can even lead to the improvement of commercial traits. For instance, the knock‐out of the tomato *PL* gene or simultaneous silencing of *PG* and *Exp1* involved in PCW disassembly reduced tomato susceptibility to *B. cinerea* and markedly extended fruit shelf‐life (Cantu et al. 2008; López‐Casado et al. 2023; Ortega‐Salazar et al. 2024; Silva et al. 2021; Wang et al. 2019). In addition, a recent study in tomato showed that reduced susceptibility to *B. cinerea* through mutation of *SDG33* and *SDG34*, involved in epigenetic regulation, improved drought tolerance without compromising plant growth (Bvindi et al. 2022).

Till now, different approaches have proven successful in overcoming pleiotropic effects caused by mutations in *S*‐genes. The most applicable one is by exploring natural and induced mutant *S*‐gene alleles (Gao et al. 2024). Loss of function of a rice *S*‐gene *ROD1*, which promotes ROS scavenging, reduced susceptibility to *R. solani* but caused lower seed production (Gao et al. 2021). Interestingly, introducing a natural *ROD1* allele (consisting of an A‐to‐C substitution in its coding sequence) with weaker activity into the *rod1* mutant alleviated the yield penalty without compromising resistance (Gao et al. 2021). Pleiotropic effects associated with induced mutant *S‐*gene alleles may be dependent on the genetic background. Impairment of *PUB17*, an *S*‐gene regulating PCD in tomato, led to the development of spontaneous necrosis on leaves in one genetic background but not in another (Ramírez Gaona et al. 2023).

The other promising approach is gene editing. Loss‐of‐function mutants of *DND1* in tomato exhibited strong growth defects, including dwarfness and auto‐necrosis (Sun et al. 2017). Surprisingly, tomato plants homozygous for a CRISPR/Cas9‐edited *DND1* gene harbouring an in‐frame deletion of 3 amino acids displayed low fitness costs while retaining the disease resistance (Li et al. 2024).

## 12 Future Gazing: The Next Frontier for *S*‐Genes

The future application of *S*‐genes in crop breeding presents significant opportunities and notable challenges. Opportunities are given by the fact that *S*‐genes are often conserved across plant species and can be modified in random and targeted ways, as shown by the above examples. In breeding, different approaches have been and can be applied to exploit mutant alleles of *S*‐genes. For example, the mutant allele of the tomato *PUB17* gene was introgressed from an EMS mutant family into cultivated tomato by classical introgression (Ramírez Gaona et al. 2023). Once GMOs are permitted in breeding programmes, precise gene editing of *S*‐genes offers great potential as demonstrated by the modification of SWEET genes (Gupta et al. 2021). Alternatively, RNAi may be a better way to silence *S*‐genes that have multiple redundant homologues, or multiple alleles in polyploid crops.

Deployment of *S*‐genes in breeding programmes is challenged by several factors, such as pleiotropic effects, recessive nature of resistance conferred by impaired *S*‐gene alleles, tissue specificity and the balance within the plant defence system, especially considering the different effects on susceptibility to biotrophic and necrotrophic pathogens. Regarding pleiotropic effects, the success of *S*‐gene utilisation can significantly depend on the genetic make‐up of the progenitor, as mentioned earlier (Ramírez Gaona et al. 2023). To address this, breeders must carefully select the genetic background for *S*‐gene deployment by leveraging genomics and bioinformatics to identify cultivars with compatible genetic profiles, thereby optimising *S*‐gene functionality (Varshney et al. 2021). This process could be further refined by targeted allele mining and functional genomics, enabling breeders to match effective alleles with the appropriate genetic backgrounds, thereby increasing the overall effectiveness of *S*‐gene deployment (Acevedo‐Garcia et al. 2014; Pavan et al. 2010; Selvakumar et al. 2023).

The recessive inheritance of *S*‐gene‐derived resistance can be resolved by using in‐gene markers for marker‐assisted selection in classical introgression breeding. For polyploid crops, selecting for desirable traits with recessive inheritance becomes more complex (van Schie and Takken 2014), but is feasible, as shown for the *mlo* mutant in wheat (Li et al. 2022). On the other hand, the genetic redundancy provided by polyploidy allows for more complex manipulations without compromising plant viability (Morineau et al. 2017). For instance, polyploid crops can harbour multiple edited versions of an *S*‐gene, providing robust resistance to pathogens while minimising negative impact on growth (Lee, Heo, et al. 2023; Shan et al. 2018). This approach not only simplifies the selection process in breeding programmes but also enables the rapid deployment of resistance traits across diverse crop species.

Finally, synthetic biology approaches such as CRISPR‐guided gene editing or prime editing combined with Artificial Intelligence (AI)‐predicted protein interactions offer promising opportunities to engineer S‐genes in such a way that their function in normal plant development is maintained, but they cannot be exploited anymore by pathogens to achieve successful infection. Although these technologies are still in early development, recent advances in plant synthetic biology (Chen et al. 2017; Khalil and Collins 2010; Pixley et al. 2019; Qi et al. 2013) suggest that practical applications could emerge within the next 5–10 years, providing a powerful tool to breed for resistance against necrotrophic pathogens.

## Author Contributions

Y.B. conceived the idea for a review paper. Y.Y. and M.R.G. wrote the manuscript. Y.B., J.A.L.v.K. and A.‐M.A.W. read and gave comments on the manuscript.

## Conflicts of Interest

The authors declare no conflicts of interest.

## Supporting information


**Table S1:** pbi70331‐sup‐0001‐TableS1.pdf. *S*‐genes mediating susceptibility to necrotrophic fungal pathogens identified in *Arabidopsis*.

## Data Availability

The authors have nothing to report.
