# Supplementary material for: Winning by Losing: Exploiting Modified Plant Susceptibility Genes to Counteract Necrotrophic Fungal Pathogens
Source: Plant Biotechnol J. 2025 Aug 25;23(12):5710–27. doi: 10.1111/pbi.70331 (PMC12665072; doi:10.1111/pbi.70331)
Supplement: Supplementary file 1 — Table S1: pbi70331‐sup‐0001‐TableS1.pdf. S‐genes mediating susceptibility to necrotrophic fungal pathogens identified in Arabidopsis. [file PBI-23-5710-s001.pdf]

Table S1. *S*-genes mediating susceptibility to necrotrophic fungal pathogens identified in *Arabidopsis*

Protein/molecule	Gene	Pathogen(s)	Function	Reference(s)
**Cuticle formation**				
Cytochrome P450	*ATT1*	*B. cinerea*	Participates in cutin biosynthesis	Tang et al. (2007)
α/β-hydrolase fold protein	*BDG*	*B. cinerea*	Participates in biosynthesis of the cutin polyester	Chassot et al. (2007); Jakobson et al. (2016)
Cytochrome P450	*LCR/LACETATE*	*B. cinerea*	Catalyzes the ω-hydroxylation of fatty acids	Bessire et al. (2007); Wellesen et al. (2001)
Long-chain acyl-CoA synthetase	*BRE1/LACS2/ SMA4*	*B. cinerea*, *S. sclerotiorum*	Catalyzes the synthesis of omega-hydroxy fatty acyl-CoA	Bessire et al. (2007); Schnurr et al. (2004); Tang et al. (2007)
ABC transporter	*PEC1/ABCG32*	*B. cinerea*	Exports cutin precursors	Bessire et al. (2011)
3-ketoacyl-CoA synthase	*FDH*	*B. cinerea*	Catalyzes synthesis of long-chain lipids	Voisin et al. (2009)
-	*ECA2*	*B. cinerea*	Participates in cutin and wax biosynthesis	Blanc et al. (2018)
Farnesyltransferase	*ERA1*	*B. cinerea*	Participates in ABA signaling and cuticle formation	Cui et al. (2019)
MYB TF	*MYB96*	*B. cinerea*	Transcriptionally activates wax biosynthetic genes	Benikhlef et al. (2013)
Cyclin-dependent kinase	*CDK8*	*B. cinerea*	Positively regulates cuticular wax biosynthesis via interaction with WAX INDUCER1, positively regulates JA-mediated defense via interaction with MED8	Zhu et al. (2014)
Defensin	*PDF1.4, PDF1.5*	*B. cinerea*	Positively regulates cuticle formation	Nguyen et al. (2023)
**Plant cell wall remodelling**				
Pectin methylesterase	*PME3*	*B. cinerea*	Catalyzes the hydrolysis of pectin to pectic acid	Bethke et al. (2014); Raiola et al. (2011)
-	*RWA2*	*B. cinerea*	Participates in PCW acetylation	Chiniquy et al. (2019); Manabe et al. (2011)
MYB TF	*MYB46*	*B. cinerea*	Regulates cellulose biosynthesis	Ramírez et al. (2011a, 2011b)
Cellulose synthase	*CESA4/IRX5, CESA7/IRX3, CESA8/IRX1*	*B. cinerea*, *Plectosphaerella cucumerina*	Participates in plant secondary cell wall formation	Hernández-Blanco et al. (2007)
Expansin-like A2 protein	*EXLA2*	*B. cinerea*	PCW-loosening agent	Abuqamar et al. (2013)
Glycine-rich protein	*GRP-3*	*B. cinerea*	Negatively regulates DAMPs-induced defense responses via interaction with WAK1	Gramegna et al. (2016)
Kinase-associated protein phosphatase	*KAPP*	*B. cinerea*	Negatively regulates DAMPs-induced defense responses via interaction with WAK1	Gramegna et al. (2016)
Cytochrome b	*AIR12*	*B. cinerea*	Participates in PCW remodelling, interacts with redox-active compounds of the apoplast	Costa et al. (2015)
Type-A response regulator	*ARR6*	*P. cucumerina*	Modulates PCW composition	Bacete et al. (2020)
**Plant cell death regulation**				
Cyclic nucleotide-gated ion channel	DND1/CNGC2	*Botrytis cinerea, Sclerotinia sclerotiorum*	Mediates Ca2+ influx into cytosol, required for PCD in plant	Clough et al. (2000); Govrin and Levine, (2000)
Papain-like cysteine protease	*RD21*	*B. cinerea, S.sclerotiorum*	Promotes oxalic acid-induced PCD in plant	Lampl et al. (2013)
NLR protein	*LAZ5*	*S. sclerotiorum*	Positively regulates PCD in plant	Barbacci et al. (2020)
LRR protein	*NTCD4*	*B. cinerea*	Promotes NLP oligomerization, plant PCD induction	Chen et al. (2021)
Class III peroxidase	*PRX33*	*Alternaria brassicicola*	Mediates ROS production and promotes PCD in plant	Kámán-Tóth et al. (2019)
Dihydrosphingosine-1-phosphate lyase	*DPL1*	*B. cinerea*	Regulates long-chain based phosphate homeostasis	Magnin-Robert et al. (2015)
Lipid acyl hydrolase	*PLP2*	*B. cinerea*	Potentiates plant PCD inflicted by necrotrophic pathogens	La Camera et al. (2005); La Camera et al. (2009)
LRR RLK	*BAK1*	*B. cinerea*, *A. brassicicola*	Phosphorylates ATG18a and suppresses autophagy	Zhang et al. (2021)
NLR protein	*LOV1*	*Cochliobolus victoriae*	Guards a defense-associated thioredoxin TRX-h5 which is targeted by victorin	Lorang et al. (2007, 2012)
**Fungal nutrient availability**				
Sugar transporter	*SWEET4*	*B. cinerea*	Mediates efflux of glucose during fungal colonization	Chong et al., (2014)
**Crosstalk of phytohormone-mediated signalling pathways and suppression of basal immunity**				
bHLH TF	*JIN1/MYC2*	*B. cinerea*, *P. cucumerina*	Negatively regulates ABA signaling, represses JA-mediated defense	Fernández-Calvo et al. (2011); Lorenzo et al. (2004)
Threonine deaminase	*OMR1*	*B. cinerea*	Participates in the synthesis of JA-isoleucine	Li et al. (2021)
LysM-containing RLK	*LYK3*	*B. cinerea*	Suppresses ABA-mediated basal defense	Paparella et al. (2014)
WRKY TF	*WRKY57*	*B. cinerea*	Negatively regulates JA signaling, transcriptional activates the repressor genes *JAZ1* and *JAZ5*	Jiang and Yu, (2016)
Homeodomain TF	*OCP3*	*B. cinerea*, *P. cucumerina*	Negatively regulates defense response mediated by ABA and JA	Coego et al. (2005); García-Andrade et al. (2011)
ERF/AP2 TF	*ERF9*	*B. cinerea*	Represses *PDF1.2* expression by binding to promoter	Maruyama et al. (2013)

Glutaredoxin	*ATGRXS13*	*B. cinerea*	Exact functions await elucidation	La Camera et al. (2011)
VQ-motif protein	*VQ22/JAV1*	*B. cinerea*	Negatively regulates JA-mediated defense, forms complex with JAZ8 and WRKY51	Hu et al. (2013)
VQ-motif protein	*VQ12, VQ29*	*B. cinerea*	Exact functions await elucidation	Wang et al. (2015)
Zeaxanthin epoxidase	*ABA1*	*P. cucumerina*	Catalyzes the first step of ABA biosynthesis	Sánchez-Vallet et al. (2012); Niyogi et al. (1998)
PYR/PYL/RCAR family protein	*PYR1, PYL1, PYL2, PYL4*	*P. cucumerina*	ABA sensor, participates in ABA signaling	García-Andrade et al. (2020); Sánchez-Vallet et al. (2012)
ERF TF	*ERF014*	*B. cinerea*	Participates in SA-mediated defense, positively regulates pectin biosynthesis	Zhang et al. (2016)
NAC TF	*ANAC019, ANAC055*	*B. cinerea*	Negatively regulate JA-mediated defense, acts downstream of MYC2	Bu et al. (2008)
NAC TF	*ATAF1*	*B. cinerea, A. brassicicola*	Negatively regulates SA- and JA-mediated defense	Wang et al. (2009)
9-cis-epoxycarotenoid dioxygenases	*NCED3, NCED5*	*B. cinerea*	Participates in ABA biosynthesis, negatively regulated by WRKY33	Liu et al. (2015)
MYB TF	*AS1*	*B. cinerea*	Positively regulates SA signlaing but negatively regulates JA-mediated defense	Nurmberg et al. (2007)
WRKY TF	*WRKY54, WRKY70*	*B. cinerea*	Negatively regulates SA biosynthesis	Li et al. (2017)
IAA-amido synthase	*GH3.2*	*B. cinerea*	Conjugates amino acids to auxin	González-Lamothe et al. (2012); Staswick et al. (2005)
RNA polymerase II CTD phosphatase	*CPL1*	*A. brassicicola*	Exact functions await elucidation	Thatcher et al. (2018)
Transmembrane protein	*RST1*	*B. cinerea, A. brassicicola*	Exact functions await elucidation	Mang et al. (2009)
DNA-binding protein	*AHL13*	*B. cinerea*	Negatively regulates JA-mediated defense	Rayapuram et al. (2021)
-	*IOP1*	*B. cinerea, A. brassicicola, P. cucumerina*	Exact functions await elucidation	Penninckx et al. (2003)
Heat shock factor	*HsfB1, HsfB2*	*A. brassicicola*	Exact functions await elucidation	Kumar et al. (2009)
F-box protein	*SLY1-10/SLEEPY1*	*A. brassicicola*	Positively regulates GA signaling, mediates degradation of GA signaling repressors DELLA	Ariizumi et al. (2011); Navarro et al. (2008)
Ent-copalyl diphosphate synthase	*GA1-3*	*A. brassicicola*	Catalyzes the conversion of geranylgeranyl pyrophosphate to copalyl pyrophosphate, participates in GA signaling	Navarro et al. (2008)
MYB TF	*MYB44*	*A. brassicicola*	Activates SA-mediated defense and represses JA-mediated defenses through direct control of WRKY70	Shim et al. (2013)
bHLH TF	*bHLH3, bHLH13, bHLH14, bHLH17*	*B. cinerea*	Negatively regulates JA-mediated defense	Song et al. (2013)
2-oxoglutarate/Fe(II)-dependent oxygenase	*JAO2*	*B. cinerea*	Hydroxylates JA to 12-OH-JA, negatively regulates JA signaling	Caarls et al. (2017); Marquis et al. (2022); Smirnova et al. (2017)
MAPKKK	*STY8, STY17, STY46*	*C. victoriae*	Negatively regulates chitin and JA signaling	Chen et al. (2024)
Spermine synthase	*SPMS*	*B. cinerea*	Synthesizes spermine, mediates JA and SA signaling	Zhang et al. (2023)
**Epigenetic regulation and post-transcriptional silencing**				
DNA glycosylase/lyase	*ROS1*	*A. brassicicola, P. cucumerina*	Mediates DNA demethylation	Gong et al. (2002); López Sánchez et al. (2016)
Linker H1 histone	*H1.1, H1.2*	*B. cinerea*	Participates in formation of compact heterochromatin domains	Sheikh et al. (2023)
miRNA	miR773	*P. cucumerina*	Targets MET2, negatively regulates plant immunity	Salvador-Guirao et al. (2018)
miRNA	miR396	*B. cinerea, P. cucumerina*	Negatively regulates a sub-set of TF genes involved in plant development and stress response	Soto-Suárez et al. (2017)
miRNA	miR396	*P. cucumerina*	Negatively regulates plant immunity	Camargo-Ramírez et al. (2018)
Argonaute protein	*AGO1*	*B. cinerea*	Major component in RNA-induced silencing complex, binds to fungal sRNAs to suppress plant immunity	Sehki et al. (2023); Weiberg et al. (2013)
SUMO protease	*SPF1, SPF2*	*B. cinerea*	Represses defense by deSUMOylation of WRKY33	Verma et a. (2021)

ABA, abscisic acid; DAMPs, damage-associated molecular pattern molecules; GA, gibberellic acid; JA, jasmonic acid; LRR, leucine-rich repeat; NLR, nucleotide-binding domain, leucine-rich repeat containing protein; MAPKKK, Mitogen Activated Protein (MAP) kinase kinase kinase; NLP, Necrosis- and ethylene-inducing peptide 1 (Nep1)-like protein; PCD, programmed cell death; PCW, plant cell wall; RLK, receptor-like protein kinase; SA, salicylic acid; TF, transcription factor.

References

Abu Qamar S, Ajeb S, Sham A, Enan MR, Iratni R (2013) A mutation in the *expansin-like A2* gene enhances resistance to necrotrophic fungi and hypersensitivity to abiotic stress in *Arabidopsis thaliana*. *Molecular Plant Pathology* **14**: 813-827.

Ariizumi T, Lawrence PK, Steber CM (2011) The role of two F-box proteins, SLEEPY1 and SNEEZY, in Arabidopsis gibberellin signaling. *Plant Physiology* **155**: 765-775.

Bacete L, Mélida H, López G, Dabos P, Tremousaygue D, Denancé N, Miedes E, Bulone V, Goffner D, Molina A (2020) Arabidopsis response regulator 6 (ARR6) modulates plant cell-wall composition and disease resistance. *Molecular Plant-Microbe Interactions* **33**: 767-780.

Barbacci A, Navaud O, Mbengue M, Barascud M, Godiard L, Khafif M, Lacaze A, Raffaele S (2020) Rapid identification of an Arabidopsis NLR gene as a candidate conferring susceptibility to *Sclerotinia sclerotiorum* using time-resolved automated phenotyping. *The Plant Journal* **103**: 903-917.

Benikhlef L, L'Haridon F, Abou-Mansour E, Serrano M, Binda M, Costa A, Lehmann S, Métraux JP (2013) Perception of soft mechanical stress in Arabidopsis leaves activates disease resistance. *BMC Plant Biology* **13**: 133.

Bessire M, Borel S, Fabre G, Carraca L, Efremova N, Yephremov A, Cao Y, Jetter R, Jacquat AC, Métraux JP, Nawrath C (2011) A member of the PLEIOTROPIC DRUG RESISTANCE family of ATP binding cassette transporters is required for the formation of a functional cuticle in Arabidopsis. *The Plant Cell* **23**: 1958-1970.

Bessire M, Chassot C, Jacquat AC, Humphry M, Borel S, MacDonald-Comber Petétot J, Métraux JP, Nawrath C (2007) A permeable cuticle in *Arabidopsis* leads to a strong resistance to *Botrytis cinerea. The EMBO journal* **26**: 2158-2168.

Bethke G, Grundman RE, Sreekanta S, Truman W, Katagiri F, Glazebrook J (2014) Arabidopsis PECTIN METHYLESTERASEs contribute to immunity against *Pseudomonas syringae*. *Plant Physiology* **164**: 1093-1107.

Blanc C, Coluccia F, L'Haridon F, Torres M, Ortiz-Berrocal M, Stahl E, Reymond P, Schreiber L, Nawrath C, Métraux JP, Serrano M (2018) The cuticle mutant *eca2* modifies plant defense responses to biotrophic and necrotrophic pathogens and herbivory insects. *Molecular Plant-Microbe Interactions* **31**: 344-355.

Bu Q, Jiang H, Li CB, Zhai Q, Zhang J, Wu X, Sun J, Xie Q, Li C (2008) Role of the *Arabidopsis thaliana* NAC transcription factors ANAC019 and ANAC055 in regulating jasmonic acid-signaled defense responses. *Cell Research* **18**: 756-767.

Caarls L, Elberse J, Awwanah M, Ludwig NR, De Vries M, Zeilmaker T, Van Wees SC, Schuurink RC, Van den Ackerveken G (2017) Arabidopsis JASMONATE-INDUCED OXYGENASES down-regulate plant immunity by hydroxylation and inactivation of the hormone jasmonic acid. *Proceedings of the National Academy of Sciences*, *USA* **114**: 6388-6393.

Camargo-Ramírez R, Val-Torregrosa B, San Segundo B (2018) MiR858-mediated regulation of flavonoid-specific MYB transcription factor genes controls resistance to pathogen infection in Arabidopsis. *Plant and Cell Physiology* **59**: 190-204.

Chassot C, Nawrath C, Métraux JP (2007) Cuticular defects lead to full immunity to a major plant pathogen. *The Plant Journal* **49**: 972-980.

Chen JB, Bao SW, Fang YL, Wei LY, Zhu WS, Peng YL, Fan J (2021) An LRR-only protein promotes NLP-triggered cell death and disease susceptibility by facilitating oligomerization of NLP in Arabidopsis. *New Phytologist* **232**: 1808-1822.

Chen L, Xiao J, Li Y, Song Y, Liu J, Zhou Q, Sun T, Wang HB, Liu B (2024) The Raf-like MAPKKKs STY8, STY17, and STY46 negatively regulate *Botrytis cinerea* resistance by limiting MKK7 protein accumulation in Arabidopsis. *The Plant Journal* **117**: 1503-1516.

Chiniquy D, Underwood W, Corwin J, Ryan A, Szemenyei H, Lim CC, Stonebloom SH, Birdseye DS, Vogel J, Kliebenstein D, Scheller HV (2019) PMR 5, an acetylation protein at the intersection of pectin biosynthesis and defense against fungal pathogens. *The Plant Journal* **100**: 1022-1035.

Chong J, Piron MC, Meyer S, Merdinoglu D, Bertsch C, Mestre P (2014) The SWEET family of sugar transporters in grapevine: VvSWEET4 is involved in the interaction with *Botrytis cinerea*. *Journal of Experimental Botany* 65: 6589-6601.

Clough SJ, Fengler KA, Yu I, Lippok B, Smith RK, Bent AF (2000) The Arabidopsis *dnd1* "defense, no death" gene encodes a mutated cyclic nucleotide-gated ion channel. *Proceedings of the National Academy of Sciences*, *USA* **97**: 9323-9328.

Coego A, Ramirez V, Gil MJ, Flors V, Mauch-Mani B, Vera P (2005) An Arabidopsis homeodomain transcription factor, *OVEREXPRESSOR OF CATIONIC PEROXIDASE* $_3$, mediates resistance to infection by necrotrophic pathogens. *The Plant Cell* **17**: 2123-2137.

Costa A, Barbaro MR, Sicilia F, Preger V, Krieger-Liszkay A, Sparla F, De Lorenzo G, Trost P (2015) AIR12, a *b*-type cytochrome of the plasma membrane of *Arabidopsis thaliana* is a negative regulator of resistance against *Botrytis cinerea*. *Plant Science* **233**: 32-43.

Cui F, Wu W, Wang K, Zhang Y, Hu Z, Brosché M, Liu S, Overmyer K (2019) Cell death regulation but not abscisic acid signaling is required for enhanced immunity to *Botrytis* in Arabidopsis cuticle-permeable mutants. *Journal of Experimental Botany* **70**: 5971-5984.

Fernández-Calvo P, Chini A, Fernández-Barbero G, Chico JM, Gimenez-Ibanez S, Geerinck J, Eeckhout D, Schweizer F, Godoy M, Franco-Zorrilla JM, Pauwels L (2011) The *Arabidopsis* bHLH transcription factors MYC3 and MYC4 are targets of JAZ repressors and act additively with MYC2 in the activation of jasmonate responses. *The Plant Cell* **23**: 701-715.

García-Andrade J, González B, Gonzalez-Guzman M, Rodriguez PL, Vera P (2020) The role of ABA in plant immunity is mediated through the PYR1 receptor. *International Journal of Molecular Sciences*. **21**: 5852.

García-Andrade J, Ramírez V, Flors V, Vera P (2011) Arabidopsis *ocp3* mutant reveals a mechanism linking ABA and JA to pathogen-induced callose deposition. *The Plant Journal* **67**: 783-794.

Gong Z, Morales-Ruiz T, Ariza RR, Roldán-Arjona T, David L, Zhu JK (2002) ROS1, a repressor of transcriptional gene silencing in *Arabidopsis*, encodes a DNA glycosylase/lyase. *Cell* **111**: 803-814.

González-Lamothe R, El Oirdi M, Brisson N, Bouarab K (2012) The conjugated auxin indole-3-acetic acid–aspartic acid promotes plant disease development. *The Plant Cell* **24**: 762-777.

Govrin EM, Levine A (2000) The hypersensitive response facilitates plant infection by the necrotrophic pathogen *Botrytis cinerea*. *Current Biology* **10**: 751-757.

Gramegna G, Modesti V, Savatin DV, Sicilia F, Cervone F, De Lorenzo G (2016) *GRP-3* and *KAPP*, encoding interactors of WAK1, negatively affect defense responses induced by oligogalacturonides and local response to wounding. *Journal of Experimental Botany* **67**: 1715-1729.

Hernández-Blanco C, Feng D, Hu J, Sánchez-Vallet A, Deslandes L, Llorente F, Berrocal-Lobo M, Keller H, Barlet X, Sánchez-Rodríguez C *et al*. (2007) Impairment of cellulose synthases required for *Arabidopsis* secondary cell wall formation enhances disease resistance. *The Plant Cell* **19**: 890-903.

Hu P, Zhou W, Cheng Z, Fan M, Wang L, Xie D (2013) JAV1 controls jasmonate-regulated plant defense. *Molecular Cell* **50**: 504-515.

Jakobson L, Lindgren LO, Verdier G, Laanemets K, Brosché M, Beisson F, Kollist H (2016) BODYGUARD is required for the biosynthesis of cutin in Arabidopsis. *New Phytologist* **211**: 614-626.

Jiang Y, Yu D (2016) The WRKY57 transcription factor affects the expression of jasmonate ZIM-domain genes transcriptionally to compromise *Botrytis cinerea* resistance. *Plant Physiology* **171**: 2771-2782.

Kámán-Tóth E, Dankó T, Gullner G, Bozsó Z, Palkovics L, Pogány M (2019) Contribution of cell wall peroxidase-and NADPH oxidase-derived reactive oxygen species to *Alternaria brassicicola*-induced oxidative burst in Arabidopsis. *Molecular Plant Pathology* **20**: 485-499.

Kumar M, Busch W, Birke H, Kemmerling B, Nürnberger T, Schöffl F (2009) Heat shock factors HsfB1 and HsfB2b are involved in the regulation of *Pdf1.2* expression and pathogen resistance in Arabidopsis. *Molecular Plant* **2**: 152-165.

La Camera S, Balagué C, Göbel C, Geoffroy P, Legrand M, Feussner I, Roby D, Heitz T (2009) The Arabidopsis patatin-like protein 2 (PLP2) plays an essential role in cell death execution and differentially affects biosynthesis of oxylipins and resistance to pathogens. *Molecular Plant-Microbe Interactions* **22**: 469-481.

La Camera S, Geoffroy P, Samaha H, Ndiaye A, Rahim G, Legrand M, Heitz T (2005) A pathogen-inducible patatin-like lipid acyl hydrolase facilitates fungal and bacterial host colonization in *Arabidopsis*. *The Plant Journal* **44**: 810-825.

La Camera S, L'Haridon F, Astier J, Zander M, Abou-Mansour E, Page G, Thurow C, Wendehenne D, Gatz C, Métraux JP, Lamotte O (2011) The glutaredoxin ATGRXS13 is required to facilitate *Botrytis cinerea* infection of *Arabidopsis thaliana* plants. *The Plant Journal* **68**: 507-519.

Lampl N, Alkan N, Davydov O, Fluhr R (2013) Set-point control of RD21 protease activity by AtSerpin1 controls cell death in Arabidopsis. *The Plant Journal* **74**: 498-510.

Li J, Zhong R, Palva ET (2017) WRKY70 and its homolog WRKY54 negatively modulate the cell wall-associated defenses to necrotrophic pathogens in Arabidopsis. *PLoS One* **12**: e0183731.

Li R, Wang L, Li Y, Zhao R, Zhang Y, Sheng J, Ma P, Shen L (2020) Knockout of *SlNPR1* enhances tomato plants resistance against *Botrytis cinerea* by modulating ROS homeostasis and JA/ET signaling pathways. *Physiologia Plantarum* **170**: 569-579.

Li Y, Li S, Du R, Wang J, Li H, Xie D, Yan J (2021) Isoleucine enhances plant resistance against *Botrytis cinerea* via jasmonate signaling pathway. *Frontiers in Plant Science* **12**: 628328.

Liu SA, Kracher B, Ziegler J, Birkenbihl RP, Somssich IE (2015) Negative regulation of ABA signaling by WRKY33 is critical for *Arabidopsis* immunity towards *Botrytis cinerea* 2100. *eLife* **4**: e07295.

López Sánchez A, Stassen JH, Furci L, Smith LM, Ton J (2016) The role of DNA (de) methylation in immune responsiveness of Arabidopsis. *The Plant Journal* **88**: 361-374.

Lorang J, Kidarsa T, Bradford CS, Gilbert B, Curtis M, Tzeng SC, Maier CS, Wolpert TJ (2012) Tricking the guard: exploiting plant defense for disease susceptibility. *Science* **338**: 659-662.

Lorang JM, Sweat TA, Wolpert TJ (2007) Plant disease susceptibility conferred by a “resistance” gene. *Proceedings of the National Academy of Sciences, USA* **104**: 14861-14866.

Lorenzo O, Chico JM, Saénchez-Serrano JJ, Solano R (2004) JASMONATE-INSENSITIVE1 encodes a MYC transcription factor essential to discriminate between different jasmonate-regulated defense responses in Arabidopsis. *The Plant Cell* **16**: 1938-1950.

Magnin-Robert M, Le Bourse D, Markham J, Dorey S, Clement C, Baillieul F, Dhondt-Cordelier S (2015) Modifications of sphingolipid content affect tolerance to hemibiotrophic and necrotrophic pathogens by modulating plant defense responses in Arabidopsis. *Plant Physiology* **169**: 2255-2274.

Manabe Y, Nafisi M, Verhertbruggen Y, Orfila C, Gille S, Rautengarten C, Cherk C, Marcus SE, Somerville S, Pauly M, Knox JP (2011) Loss-of-function mutation of REDUCED WALL ACETYLATION2 in Arabidopsis leads to reduced cell wall acetylation and increased resistance to *Botrytis cinerea*. *Plant Physiology* **155**: 1068-1078.

Mang HG, Laluk KA, Parsons EP, Kosma DK, Cooper BR, Park HC, AbuQamar S, Boccongelli C, Miyazaki S, Consiglio F, Chilosi G (2009) The Arabidopsis *RESURRECTION1* gene regulates a novel antagonistic interaction in plant defense to biotrophs and necrotrophs. *Plant Physiology* **151**: 290-305.

Marquis V, Smirnova E, Graindorge S, Delcros P, Villette C, Zumsteg J, Heintz D, Heitz T (2022) Broad-spectrum stress tolerance conferred by suppressing jasmonate signaling attenuation in Arabidopsis JASMONIC ACID OXIDASE mutants. *The Plant Journal* **109**: 856-872.

Maruyama Y, Yamoto N, Suzuki Y, Chiba Y, Yamazaki KI, Sato T, Yamaguchi J (2013) The Arabidopsis transcriptional repressor ERF9 participates in resistance against necrotrophic fungi. *Plant Science* **213**: 79-87.

Navarro L, Bari R, Achard P, Lisón P, Nemri A, Harberd NP, Jones JD (2008) DELLAs control plant immune responses by modulating the balance of jasmonic acid and salicylic acid signaling. *Current Biology* **18**: 650-655.

Nguyen NN, Lamotte O, Alsulaiman M, Ruffel S, Krouk G, Berger N, Demolombe V, Nespoulous C, Dang TM, Aimé S, Berthomieu P (2023) Reduction in *PLANT DEFENSIN 1* expression in Arabidopsis thaliana results in increased resistance to pathogens and zinc toxicity. *Journal of Experimental Botany* **74**: 5374-5393.

Niyogi KK, Grossman AR, Björkman O (1998) Arabidopsis mutants define a central role for the xanthophyll cycle in the regulation of photosynthetic energy conversion. *The Plant Cell* **10**: 1121-1134.

Nurmberg PL, Knox KA, Yun BW, Morris PC, Shafiei R, Hudson A, Loake GJ (2007) The developmental selector *AS1* is an evolutionarily conserved regulator of the plant immune response. *Proceedings of the National Academy of Sciences, USA* **104**: 18795-18800.

Paparella C, Savatin DV, Marti L, De Lorenzo G, Ferrari S (2014) The Arabidopsis LYSIN MOTIF-CONTAINING RECEPTOR-LIKE KINASE3 regulates the cross talk between immunity and abscisic acid responses. *Plant Physiology* **165**: 262-276.

Penninckx IA, Eggermont K, Schenk PM, Van den Ackerveken G, Cammue BP, Thomma BP (2003) The Arabidopsis mutant *iop1* exhibits induced over‐expression of the plant defensin gene *PDF1.2* and enhanced pathogen resistance. *Molecular Plant Pathology* **4**: 479-486.

Ramírez V, Agorio A, Coego A, García-Andrade J, Hernández MJ, Balaguer B, Ouwerkerk PB, Zarra I, Vera P (2011a) MYB46 modulates disease susceptibility to *Botrytis cinerea* in Arabidopsis. *Plant Physiology* **155**: 1920-1935.

Ramírez V, García-Andrade J, Vera P (2011b) Enhanced disease resistance to *Botrytis cinerea* in *myb46* Arabidopsis plants is associated to an early down-regulation of *CesA* genes. *Plant Signaling & Behavior* **6**: 911-913.

Rayapuram N, Jarad M, Alhoraibi HM, Bigeard J, Abulfaraj AA, Völz R, Mariappan KG, Almeida-Trapp M, Schlöffel M, Lastrucci E, Bonhomme L (2021) Chromatin phosphoproteomics unravels a function for AT-hook motif nuclear localized protein AHL13 in PAMP-triggered immunity. *Proceedings of the National Academy of Sciences*, *USA* **118**: e2004670118.

Salvador-Guirao R, Baldrich P, Weigel D, Rubio-Somoza I, San Segundo B (2018) The microRNA miR773 is involved in the *Arabidopsis* immune response to fungal pathogens. *Molecular Plant-Microbe Interactions* **31**: 249-259.

Sánchez-Vallet A, López G, Ramos B, Delgado-Cerezo M, Riviere MP, Llorente F, Fernández PV, Miedes E, Estevez JM, Grant M, Molina A (2012) Disruption of abscisic acid signaling constitutively activates Arabidopsis resistance to the necrotrophic fungus *Plectosphaerella cucumerina*. *Plant Physiology* **160**: 2109-2124.

Schnurr J, Shockey J, Browse J (2004) The acyl-CoA synthetase encoded by *LACS2* is essential for normal cuticle development in Arabidopsis. *The Plant Cell* **16**: 629-642.

Sehki H, Yu A, Elmayan T, Vaucheret H (2023) TYMV and TRV infect *Arabidopsis thaliana* by expressing weak suppressors of RNA silencing and inducing host RNASE THREE LIKE1. *PLoS Pathogens* **19**: e1010482.

Sheikh AH, Nawaz K, Tabassum N, Almeida-Trapp M, Mariappan KG, Alhoraibi H, Rayapuram N, Aranda M, Groth M, Hirt H (2023) Linker histone H1 modulates defense priming and immunity in plants. *Nucleic Acids Research* **51**: 4252-4265.

Shim JS, Jung C, Lee S, Min K, Lee YW, Choi Y, Lee JS, Song JT, Kim JK, Choi YD (2013) *AtMYB44* regulates *WRKY70* expression and modulates antagonistic interaction between salicylic acid and jasmonic acid signaling. *The Plant Journal* **73**: 483-495.

Smirnova E, Marquis V, Poirier L, Aubert Y, Zumsteg J, Ménard R, Miesch L, Heitz T (2017) Jasmonic acid oxidase 2 hydroxylates jasmonic acid and represses basal defense and resistance responses against *Botrytis cinere* infection. *Molecular Plant* **10**: 1159-1173.

Song S, Qi T, Fan M, Zhang X, Gao H, Huang H, Wu D, Guo H, Xie D (2013) The bHLH subgroup IIId factors negatively regulate jasmonate-mediated plant defense and development. *PLoS Genetics* **9**: e1003653.

Soto-Suárez M, Baldrich P, Weigel D, Rubio-Somoza I, San Segundo B (2017) The Arabidopsis miR396 mediates pathogen-associated molecular pattern-triggered immune responses against fungal pathogens. *Scientific Reports* **7**: 44898.

Staswick PE, Serban B, Rowe M, Tiryaki I, Maldonado MT, Maldonado MC, Suza W (2005) Characterization of an Arabidopsis enzyme family that conjugates amino acids to indole-3-acetic acid. *The Plant Cell* **17**: 616-627.

Tang D, Simonich MT, Innes RW (2007) Mutations in LACS2, a long-chain acyl-coenzyme A synthetase, enhance susceptibility to avirulent *Pseudomonas syringae* but confer resistance to *Botrytis cinerea* in Arabidopsis. *Plant Physiology* **144**: 1093-1103.

Thatcher LF, Foley R, Casarotto HJ, Gao LL, Kamphuis LG, Melser S, Singh KB (2018) The Arabidopsis RNA polymerase II carboxyl terminal domain (CTD) phosphatase-like1 (CPL1) is a biotic stress susceptibility gene. *Scientific Reports* **8**: 13454.

Verma V, Srivastava AK, Gough C, Campanaro A, Srivastava M, Morrell R, Joyce J, Bailey M, Zhang C, Krysan PJ, Sadanandom A (2021) SUMO enables substrate selectivity by mitogen-activated protein kinases to regulate immunity in plants. *Proceedings of the National Academy of Sciences*, *USA* **118**: e2021351118.

Voisin D, Nawrath C, Kurdyukov S, Franke RB, Reina-Pinto JJ, Efremova N, Will I, Schreiber L, Yephremov A (2009) Dissection of the complex phenotype in cuticular mutants of Arabidopsis reveals a role of SERRATE as a mediator. *PLoS Genetics* **5**: e1000703.

Wang H, Hu Y, Pan J, Yu D (2015) Arabidopsis VQ motif-containing proteins VQ12 and VQ29 negatively modulate basal defense against *Botrytis cinerea*. *Scientific Reports* **5**: 14185.

Wang XE, Basnayake BV, Zhang H, Li G, Li W, Virk N, Mengiste T, Song F (2009) The *Arabidopsis* ATAF1, a NAC transcription factor, is a negative regulator of defense responses against necrotrophic fungal and bacterial pathogens. *Molecular Plant-Microbe Interactions* **22**: 1227-1238.

Weiberg A, Wang M, Lin FM, Zhao H, Zhang Z, Kaloshian I, Huang HD, Jin H (2013) Fungal small RNAs suppress plant immunity by hijacking host RNA interference pathways. *Science* **342**: 118-123.

Wellesen K, Durst F, Pinot F, Benveniste I, Nettesheim K, Wisman E, Steiner-Lange S, Saedler H, Yephremov A (2001) Functional analysis of the *LACERATA* gene of Arabidopsis provides evidence for different roles of fatty acid ω-hydroxylation in development. *Proceedings of the National Academy of Sciences*, *USA* **98**: 9694-9699.

Zhang C, Atanasov KE, Murillo E, Vives‐Peris V, Zhao J, Deng C, Gómez‐Cadenas A, Alcázar R (2023) Spermine deficiency shifts the balance between jasmonic acid and salicylic acid‐mediated defence responses in Arabidopsis. *Plant, Cell & Environment* **46**: 3949-3970.

Zhang B, Shao L, Wang J, Zhang Y, Guo X, Peng Y, Cao Y, Lai Z (2021) Phosphorylation of ATG18a by BAK1 suppresses autophagy and attenuates plant resistance against necrotrophic pathogens. *Autophagy* **17**: 2093-2110.

Zhang H, Hong Y, Huang L, Li D, Song F (2016) Arabidopsis AtERF014 acts as a dual regulator that differentially modulates immunity against *Pseudomonas syringae* pv. *tomato* and *Botrytis cinerea*. *Scientific Reports* **6**: 30251.

Zhu Y, Schluttenhoffer CM, Wang P, Fu F, Thimmapuram J, Zhu JK, Lee SY, Yun DJ, Mengiste T (2014) CYCLIN-DEPENDENT KINASE8 differentially regulates plant immunity to fungal pathogens through kinase-dependent and-independent functions in Arabidopsis. *The Plant Cell* **26**: 4149-4170.
